# Supplementary material for: Habitat selection of female sharp-tailed grouse in grasslands managed for livestock production
Source: PLoS One. 2020 Jun 4;15(6):e0233756. doi: 10.1371/journal.pone.0233756 (PMC7272000; doi:10.1371/journal.pone.0233756)
Supplement: S1 Appendix — (DOCX) [file pone.0233756.s001.docx]

S1 Appendix. Example code for Bayesian logistic regression model evaluating third order habitat selection using the R package R-INLA.

formula <- y ~ grass1300 + draws1300 + roaddist + StockNow + restrot + rot +

f(id, model = "iid",

hyper = list(theta = list(initial = log(1e-6), fixed = TRUE))) +

f(id.grass, grass1300, values = 1:4, model = "iid",

hyper = list(theta = list(initial = log(1), fixed = FALSE,

prior = "pc.prec", param = c(1, .05)))) +

f(id.draws, draws1300, values = 1:4, model="iid",

hyper = list(theta = list(initial = log(1), fixed = FALSE,

prior = "pc.prec", param = c(1, .05)))) +

f(id.road, roaddist, values = 1:4, model="iid",

hyper = list(theta = list(initial = log(1), fixed = FALSE,

prior = "pc.prec", param = c(1, .05)))) +

f(id.StockNow, StockNow, values = 1:4, model="iid",

hyper = list(theta = list(initial = log(1), fixed = FALSE,

prior = "pc.prec", param = c(1, .05)))) +

f(id.restrot, restrot, values = 1:4, model="iid",

hyper = list(theta = list(initial = log(1), fixed = FALSE,

prior = "pc.prec", param = c(1, .05)))) +

f(id.rot, rot, values = 1:4, model="iid",

hyper = list(theta = list(initial = log(1), fixed = FALSE,

prior = "pc.prec", param = c(1, .05))))

# inla() call

model <- inla(formula, weights=rsf$weights, family = "binomial", data = rsf, control.compute = list(dic = TRUE, waic = TRUE))
