# Supplementary figures and images for: Habitat selection of female sharp-tailed grouse in grasslands managed for livestock production
Source: PLoS One. 2020 Jun 4;15(6):e0233756. doi: 10.1371/journal.pone.0233756 (PMC7272000; doi:10.1371/journal.pone.0233756)

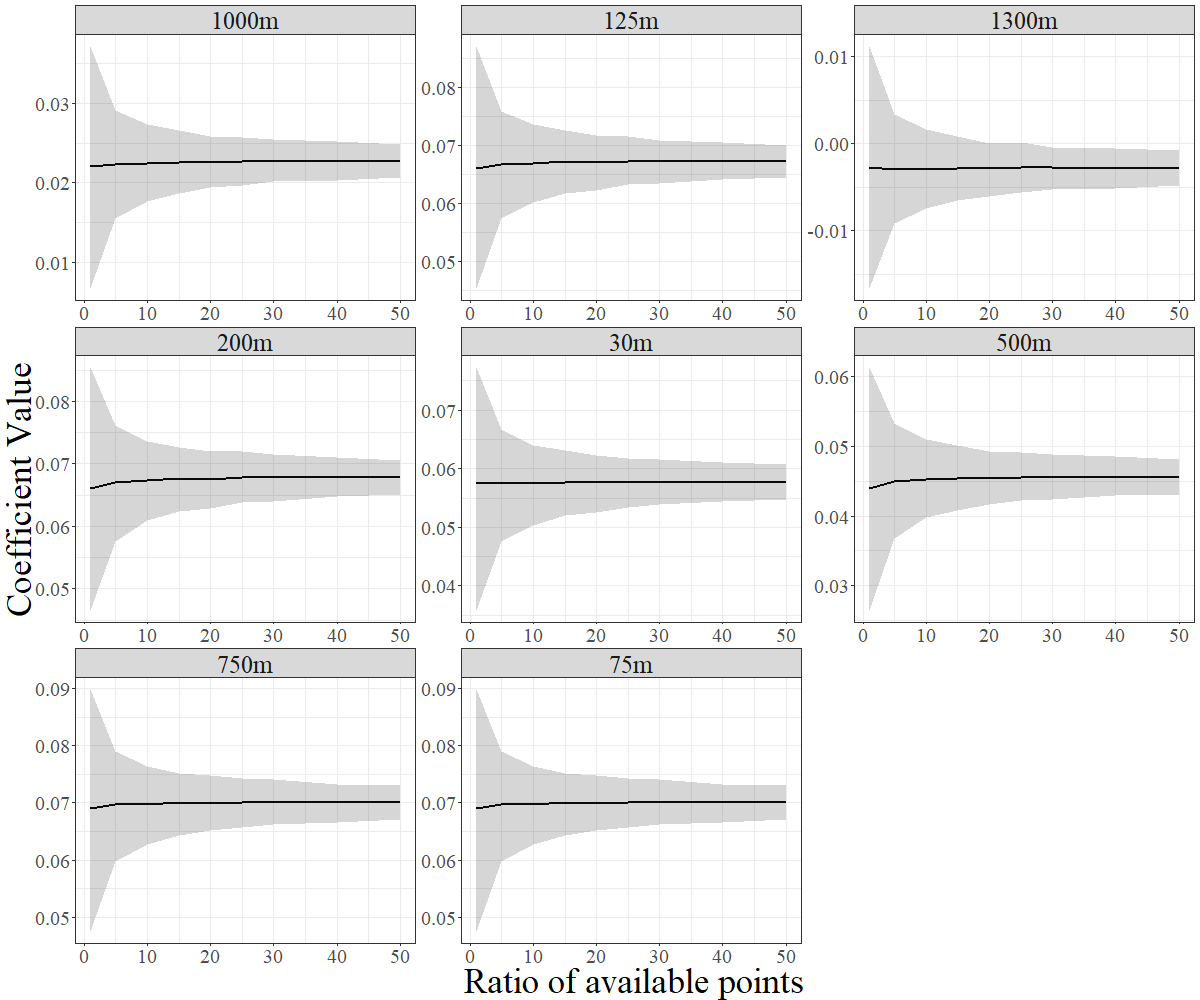

Supplement: S1 Fig — (TIF) [file pone.0233756.s002.tif]

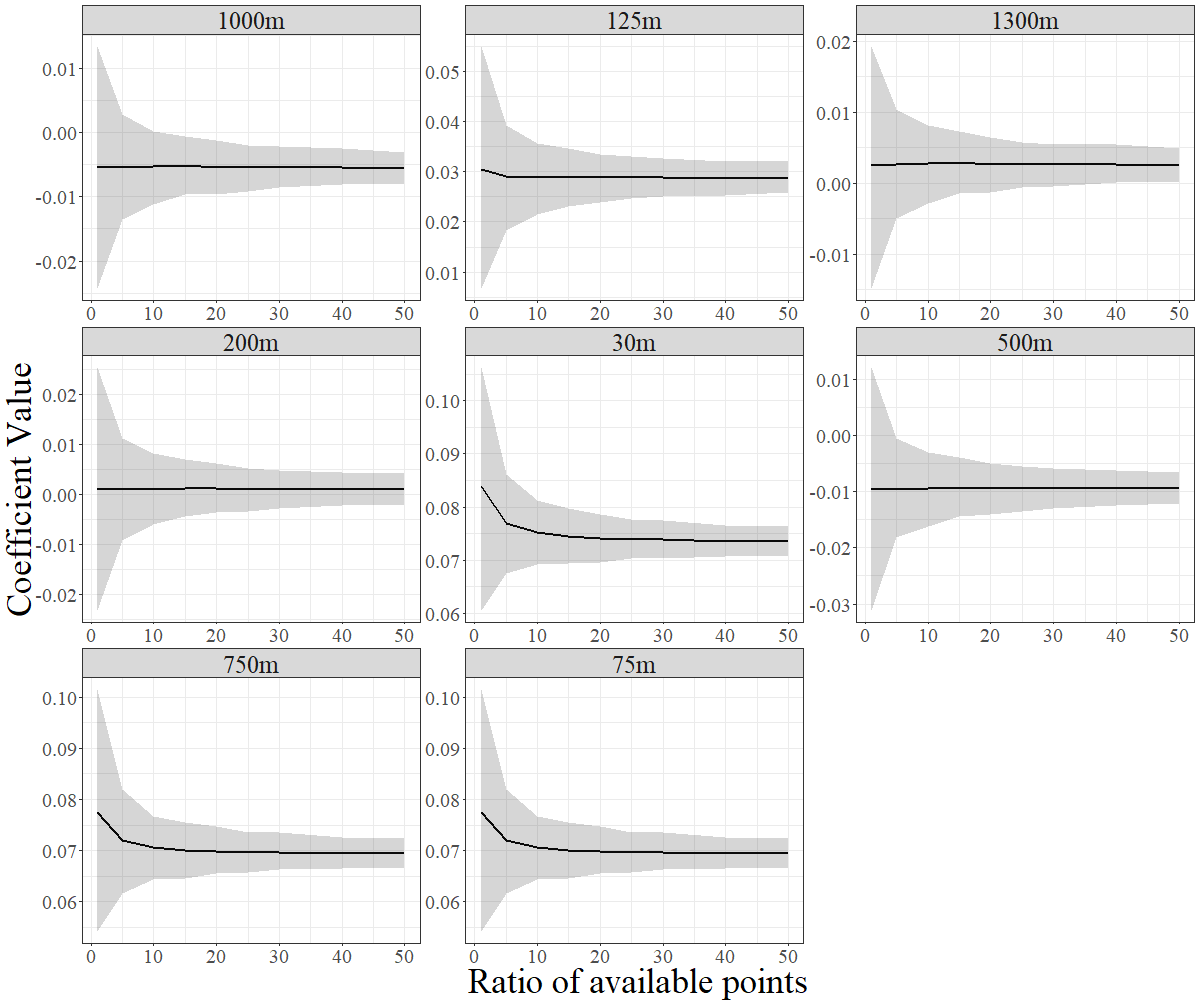

Supplement: S2 Fig — (TIF) [file pone.0233756.s003.tif]

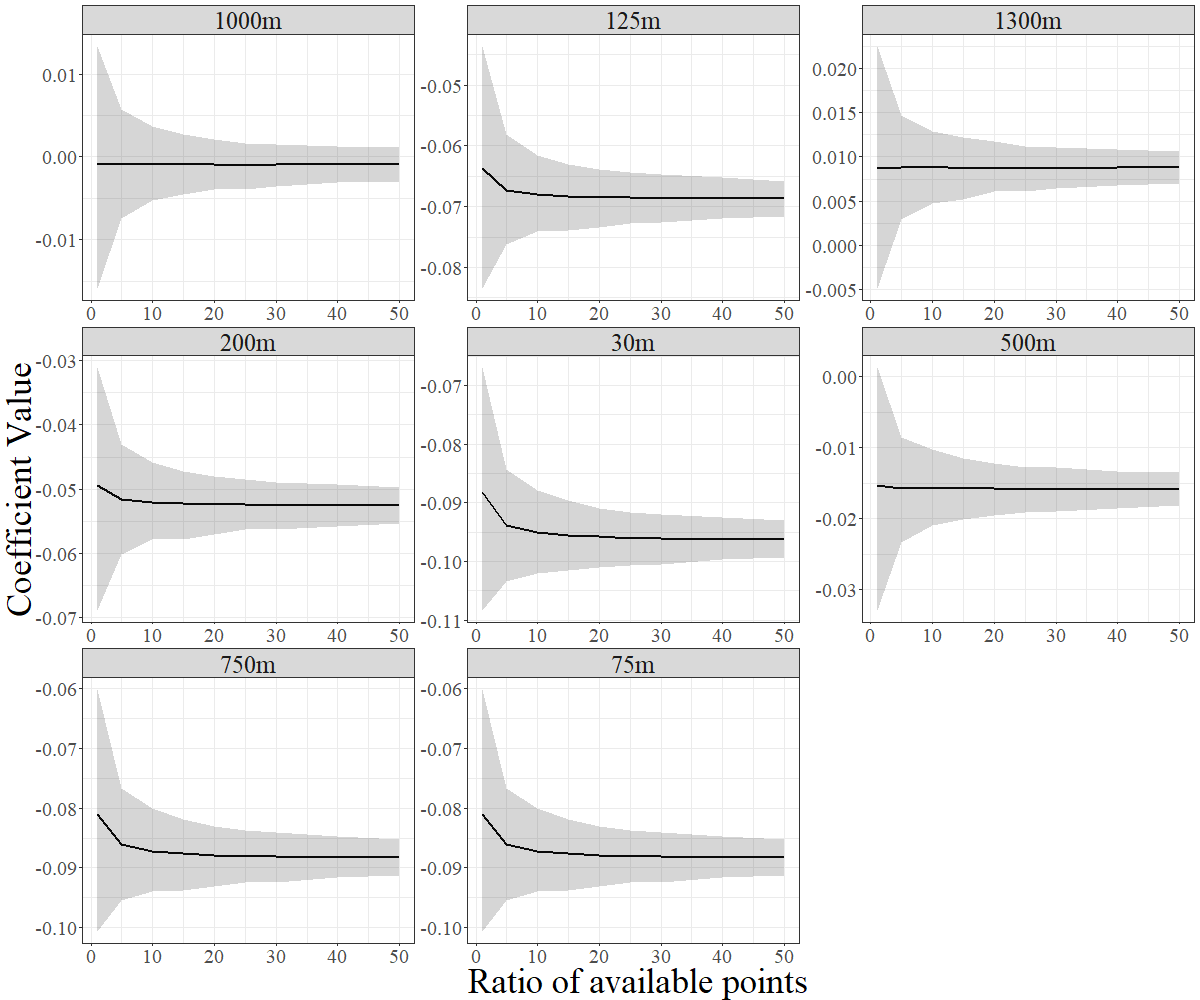

Supplement: S3 Fig — (TIF) [file pone.0233756.s004.tif]

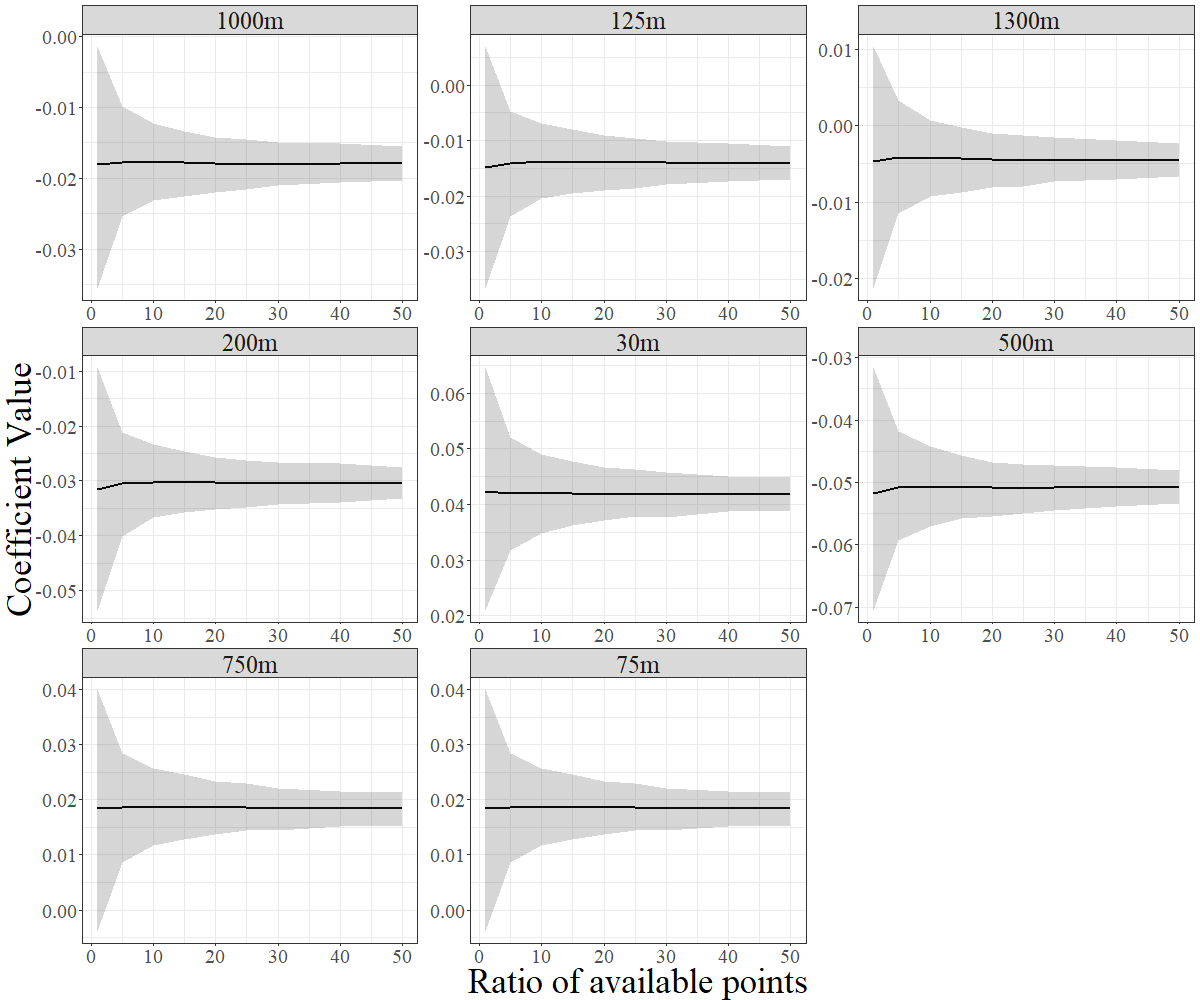

Supplement: S4 Fig — (TIF) [file pone.0233756.s005.tif]

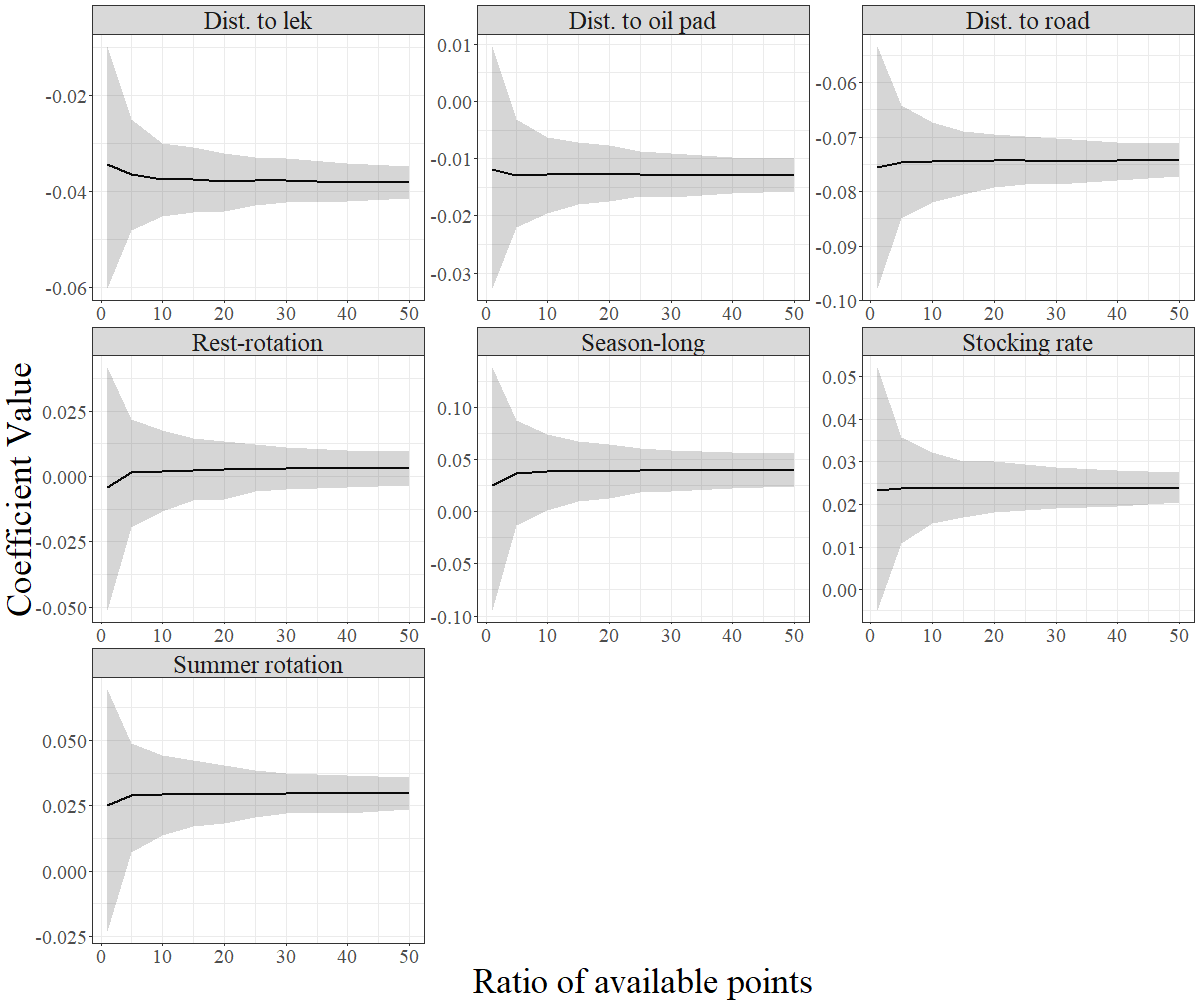

Supplement: S5 Fig — (TIF) [file pone.0233756.s006.tif]
