# Supplementary material for: Habitat selection of female sharp-tailed grouse in grasslands managed for livestock production
Source: PLoS One. 2020 Jun 4;15(6):e0233756. doi: 10.1371/journal.pone.0233756 (PMC7272000; doi:10.1371/journal.pone.0233756)
Supplement: S1 Table — The number of parameters (K), AICc values, AICc values, model weights (wi), and log-likelihoods are reported. (DOCX) [file pone.0233756.s007.docx]

| S1 Table. Support for candidate models predicting the relationship between the number of locations per female and home range size of female sharp-tailed grouse during the breeding seasons of 2016–2018. The number of parameters (K), AIC_c_ values, AIC_c_ values, model weights (*w_i_*), and log-likelihoods are reported. | | | | | | |
| --- | --- | --- | --- | --- | --- | --- |
| **Model** | **K** | **AIC_c_** | **ΔAIC_c_** | **AIC_c_ *w_i_*** | **Cum. *w_i_*** | **LogLik** |
| Null | 2 | 2166.80 | 0.00 | 0.45 | 0.45 | -1081.36 |
| # Locations | 3 | 2167.23 | 0.44 | 0.36 | 0.81 | -1080.53 |
| # Locations + # Locations^2^ | 4 | 2168.53 | 1.74 | 0.19 | 1.00 | -1080.12 |
