# Supplementary material for: Habitat selection of female sharp-tailed grouse in grasslands managed for livestock production
Source: PLoS One. 2020 Jun 4;15(6):e0233756. doi: 10.1371/journal.pone.0233756 (PMC7272000; doi:10.1371/journal.pone.0233756)
Supplement: S2 Table — The number of parameters (K), AICc values, AICc values, model weights (wi), and log-likelihoods are reported. (DOCX) [file pone.0233756.s008.docx]

| S2 Table. Support for candidate models predicting the relationship between habitat and anthropogenic variables and home range selection of female sharp-tailed grouse during the breeding seasons of 2016–2018. The number of parameters (K), AIC_c_ values, AIC_c_ values, model weights (*w_i_*), and log-likelihoods are reported. | | | | | | |
| --- | --- | --- | --- | --- | --- | --- |
| **Model** | **K** | **AIC_c_** | **ΔAIC_c_** | **AIC_c_ w_i_** | **Cum. w_i_** | **LL** |
| % Grassland + % wooded draws + dist. to oil pad | 4 | 2326.07 | 0.00 | 0.54 | 0.54 | -1159.02 |
| % Grassland + % wooded draws + dist. to oil pad + dist. to road | 5 | 2327.68 | 1.60 | 0.24 | 0.78 | -1158.81 |
| % Grassland + % wooded draws | 3 | 2329.75 | 3.68 | 0.09 | 0.86 | -1161.87 |
| % Grassland + dist. to oil pad | 3 | 2330.84 | 4.77 | 0.05 | 0.91 | -1162.41 |
| % Grassland + % wooded draws + dist. to road | 4 | 2331.70 | 5.62 | 0.03 | 0.95 | -1161.83 |
| % Grassland + dist. to oil pad + dist. to road | 4 | 2332.14 | 6.06 | 0.03 | 0.97 | -1162.05 |
| % Grassland | 2 | 2333.75 | 7.67 | 0.01 | 0.98 | -1164.87 |
| % Grassland + dist. to road | 3 | 2335.75 | 9.67 | 0.00 | 0.99 | -1164.86 |
| Null | 1 | 2336.38 | 10.31 | 0.00 | 0.99 | -1167.19 |
| % Wooded draws | 2 | 2336.81 | 10.74 | 0.00 | 0.99 | -1166.40 |
| Dist. to oil pad | 2 | 2337.81 | 11.73 | 0.00 | 0.99 | -1166.90 |
| Dist. to road | 2 | 2338.01 | 11.94 | 0.00 | 1.00 | -1167.00 |
| % Wooded draws + dist. to oil pad | 3 | 2338.46 | 12.39 | 0.00 | 1.00 | -1166.22 |
| % Wooded draws + dist. to road | 3 | 2338.53 | 12.45 | 0.00 | 1.00 | -1166.25 |
| Dist. to oil pad + dist. to road | 3 | 2338.63 | 12.55 | 0.00 | 1.00 | -1166.30 |
| % Wooded draws + dist. to oil pad + dist. to road | 4 | 2339.55 | 13.47 | 0.00 | 1.00 | -1165.75 |
