# Supplementary material for: Habitat selection of female sharp-tailed grouse in grasslands managed for livestock production
Source: PLoS One. 2020 Jun 4;15(6):e0233756. doi: 10.1371/journal.pone.0233756 (PMC7272000; doi:10.1371/journal.pone.0233756)
Supplement: S3 Table — (DOCX) [file pone.0233756.s009.docx]

| S3 Table. Support for models predicting the spatial grain of each landcover variable that best predicts sharp-tailed grouse habitat selection based during the breeding seasons of 2016–2018 on deviance information criteria (DIC) values. | | | | |
| --- | --- | --- | --- | --- |
| **Grain** | **% Grassland** | **% Wooded draws** | **% Agriculture** | **Edge density** |
| 30m | 63017.46 | 63258.08 | 63235.63 | 62875.77 |
| 75m | 63030.43 | 63152.05 | 63235.30 | 62879.39 |
| 125m | 62996.12 | 63010.50 | 63215.67 | 62826.72 |
| 200m | 62923.00 | 62873.43 | 63209.34 | 62673.41 |
| 500m | 62920.91 | 62664.49 | 63206.24 | 62632.73 |
| 750m | 62842.46 | 62747.64 | 63250.02 | 62614.43 |
| 1000m | 62853.75 | 62586.77 | 63252.53 | 62721.80 |
| 1300m | 62805.31 | 62402.51 | 63216.24 | 62760.03 |
