# Supplementary material for: Habitat selection of female sharp-tailed grouse in grasslands managed for livestock production
Source: PLoS One. 2020 Jun 4;15(6):e0233756. doi: 10.1371/journal.pone.0233756 (PMC7272000; doi:10.1371/journal.pone.0233756)
Supplement: S4 Table — (DOCX) [file pone.0233756.s010.docx]

| S4 Table. Multicollinearity results for management and landscape variables in the full third order resource selection analysis evaluating habitat selection within the home range for sharp-tailed grouse during the breeding seasons of 2016–2018. | | | | | | | |
| --- | --- | --- | --- | --- | --- | --- | --- |
|  | **%**  **Grassland** | **% Wooded draws** | **% Agriculture** | **Edge density** | **Dist. to oil pad** | **Dist. to road** | **Stocking rate (current)** |
| **% Wooded draws** | -0.54 |  |  |  |  |  |  |
| **%**  **Agriculture** | -0.72 | 0.17 |  |  |  |  |  |
| **Edge density** | -0.64 | 0.62 | 0.25 |  |  |  |  |
| **Dist. to oil pad** | -0.55 | 0.21 | 0.48 | 0.11 |  |  |  |
| **Dist. to road** | 0.48 | -0.19 | -0.36 | -0.06 | -0.35 |  |  |
| **Stocking rate (current)** | -0.08 | -0.02 | -0.05 | 0.08 | -0.02 | -0.01 |  |
| **Stocking rate (previous)** | 0.11 | -0.07 | -0.11 | -0.09 | -0.14 | 0.11 | 0.47 |
