# Supplementary material for: Habitat selection of female sharp-tailed grouse in grasslands managed for livestock production
Source: PLoS One. 2020 Jun 4;15(6):e0233756. doi: 10.1371/journal.pone.0233756 (PMC7272000; doi:10.1371/journal.pone.0233756)
Supplement: Attachment — Submitted filename: milligan sharptail nest selection review.pdf [file pone.0233756.s012.pdf]

Overall, this is a nicely set-up study that seeks to answer some pertinent, long-unanswered questions about effects of livestock management on a sharp-tailed grouse and their breeding habitat. The paper is generally well written and the conclusions appear well-supported by the results. I do, however, have a couple substantial issues with the analysis and its interpretation. These concerns are related and may be cleared up fairly quickly.

First, the third-order resource selection model seems to be misspecified. Random effects seem to have been included in a way that makes no biological or mathematical sense.

Second, the considerable space is given to interpreting these random effects as 'individual variation' in behavior, which is an interpretation that the model structure—if I'm seeing it right—doesn't support.

Overall, I'm having a hard time knowing what I'm looking at in the results because the methods lack important details about the model. I would like to see the methods clarified by including full model statements (including priors—the reader is told that the priors are vague or uninformative, but has no way of verifying that for themselves because they are not provided) and, ideally, JAGS model code in Supplemental Materials.

If I am correct in my understanding that random intercepts, but not random slopes, were included in this RSF model, then I would like to see this corrected by either a) removing the random intercepts and any references to variation among individuals in their behavior, since this wasn't a feature of the model, or b) adding random slopes and interpreting those parameters as individual variation.

Option A would take hardly any time at all, whereas Option B would entail a bit of work. I'm not set on either one, but I do tend to think a random-intercept/random-slope model is more appropriate for this study design.

Best regards,
Joe Smith

<u>Line-by-line comments:</u>

Line 25: May need to remove reference to individual-level variation in habitat selection; see detailed comments on methods below.

Line 39: I'm being nitpicky here, but this is such a vague, fluffy statement that it could mean almost anything. Perhaps a specific example of how 'conservation and management actions' could 'consider' this information would imbue this statement with some needed substance.

Line 54: Make the speculative/hypothetical nature of this claim perfectly clear here. The two provided citations provide no actual evidence that they function like patch-burn grazing. The UGBEP makes a pretty unequivocal statement that they benefit upland birds, but provides no evidence backing that up whatsoever. Frisina's paper only deals with whether rotational grazing can reduce conflict between cattle and elk on winter range, and never mentions heterogeneity. Just make sure you don't accidentally help propagate misinformation by leading the reader (most of whom don't check citations) to believe that there are studies that support this.

Line 61: Same comment here as above. Yes, they will have increased residual cover, but I don't think I've ever seen any evidence that RGS increase structural heterogeneity or create a 'patchwork' relative to other grazing systems (I haven't searched exhaustively, though). We actually found the opposite effect in Roundup, and it looks like heterogeneity in VOR was lowest on RGS ranches in your study, too (Fig 6, random plots, in your recent REM paper--you could even cite that). You could simply add a statement like, "but this hypothesis has not been adequately tested."

I think the purpose of RGS may have been lost in translation between range managers and wildlifers. In Bailey and Brown (2011) *Rangeland Ecol Manage* 64:1–9, they write, "To alleviate selective grazing across multiple scales, many rangeland managers have implemented rotational grazing systems (RGS)...The potential benefits are projected to derive from a decrease in area available to livestock at any given time (improved distribution); an increase in stock density (increased uniformity of defoliation across species and communities)..."

So, rangeland managers may be quite aware that these systems are having the exact opposite effect, at least in some settings.

Lines 70–71: This sentence is also very vague. What kind of 'implications?'

Line 185: Define an 'abrupt change.'

Line 200: I'd like to see more detail on your methods for the second-order resource selection analysis, rather than just referring the reader to a couple citations. Which variables were considered, how was model fitting and model selection accomplished? There's no way I could re-create your analysis with the details you give here.

Lines 232–234: "For all models, we used generalized linear mixed models in a Bayesian framework with a logit-link and female ID as a random intercept to account for potential autocorrelation among sampling points [62, 63]."

I'm pretty sure this isn't doing what you intended. Given your particular availability sampling design, these random intercepts are unnecessary and shouldn't affect your inference at all. You later (lines 253–255) describe σ^2 as representing individual-level variation in selection for various habitat variables, which is a bit different than the way you justify inclusion of random

intercepts here (autocorrelation in the response variable--not sure what that means in this case), but this is not what random intercepts do, either.

Consider what the intercept represents in a simple, univariate logit-link resource selection function where you've centered your covariate at zero. If you convert it to the probability scale, it's the predicted probability that a case/sample is a used case (1) rather than an available case (0) at the mean value of the covariate. In a use-availability RSF, its value is going to reflect the prevalence of use cases—your ratio of used to available samples. You determined that when you decided how many random samples to include for each used sample. The intercept has no biological meaning in an RSF, it is simply a reflection of the way the investigator sets up the analysis.

Unless you included both random intercepts *and* random adjustments to the slope (coefficient for the covariate—e.g., $\beta_j \sim N(\overline{\beta}, \sigma^2_\beta)$,where the *j* subscript indexes individuals, from your citation #69), then all you are doing by allowing the intercept to vary among individuals is allowing random adjustments to the *overall* (i.e., regardless of the covariate value) probability of use. Given this is a design parameter, not a biological one, and in your study design you've fixed it at 1:15 = 0.0625 for all individuals in your dataset (this is why your y-axis tops out at about 0.07 in Fig. 4), there is no reason to expect that there will be any variation among individuals at all. By design, individuals do not vary in mean P(use) (i.e., prevalance), and you've fixed the regression coefficients, so they don't vary in their responses to covariates. So what *is* the variation you're modeling with these random effects?

You can prove this to yourself by running this R code with your data:

```
require(here)
require(tidyr)
require(dplyr)
require(lme4)

df <- read.csv(here('data/Pts.15.FULL.csv'), stringsAsFactors = FALSE)

# check that prevalence is equal across individuals
df %>%
  select(id, use) %>%
  group_by(id) %>%
  summarize(prevalence = mean(use)) %>%
  pull(prevalence)

# scale and center continuous covariates
df.scaled <- df %>%
  mutate_if(is.double, scale)

# fit a simple univariate fixed-effects glm
```

```
m.fixed <- glm(use ~ 1 + roaddist, data=df.scaled, family=binomial(link =
'logit'))

# fit the same model, but with random intercepts for individuals
m.rand.intercept <- glmer(use ~ 1 + roaddist + (1|id), data=df.scaled,
family=binomial(link = 'logit'))
# note warning about boundary effect

# check estimates and SE's of fixed effects (your inference)
summary(m.fixed)
summary(m.rand.intercept)
```

The SEs and coefficient estimates are equal for these two model specifications. Your inference is exactly the same. Also look at the estimate of individual-level variation for the glmer--it's effectively zero (the boundary effect).

The random intercept takes on a very different role when you also include random slopes. If individuals vary in their selection for a covariate, then you need random intercepts because it doesn't make sense to force P(use) through a single point along the x-axis. If some respond positively and others negatively to distance to roads, for example, there's no reason to expect they'll all converge on the exact same probability of selection at the mean distance to roads in your dataset. Random intercepts give some needed flexibility there.

Your random intercepts, however, have no clear biological interpretation that I can think of, which is probably why your results show they're all about the same, may in fact be zero, and, if you plotted their densities instead of means and CRIs, you'd probably find them to closely reflect their priors (which I'm guessing were Uniform(0,2) based on Fig. 5).

This is a really long-winded way of saying you need random slopes, not just random intercepts, if you want to know anything about individual-level variation in selection. If you don't, you should take the random intercepts out because prevalence is a design parameter that you've fixed to be constant across individuals (Line 231). They are not affecting your inference.

Line 243: "Regression coefficients for each variable were the product of binary indicator variables and both continuous and categorical covariates."

Hmmm, this doesn't make sense the way it's worded. They should be the product of the indicator variable and an effect size parameter (the 'raw' coefficient). Multiplying the indicator by the covariate (the whole vector? I'm having a hard time picturing what you mean here) will give you either a vector of zeros or your original vector of covariates. It won't give you a regression coefficient.

Line 246: “We assumed that all variables with high inclusion probability based on the posterior distributions of their indicator variables influenced habitat selection and variables with inclusion probabilities ≤ 0.25 were unimportant [67]”

Mutshinda et al. used ≤0.25 to identify unimportant variables because they used a fixed prior probability of 0.5, so that posterior probability coincides with a Bayes factor of 0.33, or 3:1 against the variable. Because you have a distribution of prior odds, rather than a fixed value, 0.25 doesn’t have any real meaning here. If, for example, your model converged on an inclusion probability of 0.1, then 0.25 would actually represent a Bayes factor of 3, or 3:1 evidence *for* the variable. If anything, I could see taking the median posterior inclusion probability and using that to calculate the cutoff, but that seems a little circular, too.

I’d argue that variable selection is simpler if you just pick what you think is an appropriate prior probability and consider it fixed. With the amount of data you have, I doubt this value is going to have much of an influence on which variables are identified as important, but you could always try running it with a few values to check that sensitivity.

Unless mean variable inclusion probability is a parameter you’re interested in making inference on (which it doesn’t seem like it is, given that you don’t report it in the results), then I don’t quite see the value in putting a prior on it and estimating it.

Line 250: Define “standardized coefficients.”

Line 252: So are you using credible intervals or the posterior inclusion probability to determine which variables are important? Is one given higher priority than another?

Line 253: I’m not exactly sure what these σ^2 are, but given that you did not include random slopes, they cannot represent individual variation in behavior.

Line 259: “Vague uniform or normal priors were used for all model parameters related to covariates and their measures of error.”

This statement is itself a little vague. I would like to see either a) complete model statements in your methods section or b) your JAGS model code (in supplemental info?) so I can see exactly what priors were placed on each parameter. Ideally, provide both.

Line 269: Insert ‘Gelman-Rubin statistic’ before ‘values’ or just refer to them as ‘potential scale reduction factors’ so we know what values you’re referring to.

Line 270: I’m glad to see you assessed model fit, but it would be nice to know which specific ‘attribute of the data’ you used to calculate the Bayesian *p*-value.

Line 290: Were any models with >1 predictor considered? Again, more details on the 2nd order resource selection analysis are needed.

Lines 292–293: This sentence and Table 3 seem to provide exactly the same information, so I would just include one or the other. I find Table 3 hard to interpret (isn't the info on either side of the diagonal redundant? And why isn't there a #2-ranked landcover?), so I'd suggest axing it.

Line 308: Again, I don't think 0.25 is a meaningful cutoff. Try to come up with a criterion more applicable to your particular model.

Line 311: This doesn't indicate individual variation in habitat selection unless you fit random intercept and random slope models. So am I right to assume these variances indicate variation among individual-level adjustments to the intercept? If so, why is there more than one? Are these from separate, univariate models? I'm generally very confused about your model structure, so I'll reiterate here that it would really help if you provided model statements and code so readers know exactly what parameters these are.

Also (and this is a personal preference thing unless the journal has specific guidelines about it), I'm much more accustomed to thinking of variation on the scale of standard deviations rather than variances. Seems easier to interpret since it's on the same scale as the regression coefficients and data. Is there a specific reason you report σ^2 rather than σ?

Line 377: Given that you have the ability to fit random-slope, random-intercept models (because you sampled availability at the individual level), there is no reason you shouldn't. In fact, I would argue that assuming the slopes are fixed across individuals is inappropriate given that availability differed across your study area, as you state on Line 348. At the individual level and the third-order, you are only likely to detect avoidance of, say, oil and gas features, if there are oil and gas features within the home range to avoid. By forcing all the responses to be the same among individuals, you're not allowing your models to pick up on those potential functional responses to availability.

Moreover, fitting random-slope, random-intercept models does not preclude making inference at the population level. The beauty of these multi-level models is that you can make inference at multiple levels of the hierarchy—population or individual. Going back to the example of citation #69, this model provides not only estimates of the individual-level β_j's, but also the population-level $\overline{\beta}$.

Fig. 1. Might this look a little better on the log(ha) scale?

Fig. 2. Since both variables are continuous, why not plot the data? It's a more honest way of showing the strength and predictive capability of a relationship than just plotting the regression line for the mean and its confidence interval.

Fig. 3 and Fig. 5: Both of these figures depict information that seems better suited to a table. Just a suggestion.
