# Supplementary material for: Habitat selection of female sharp-tailed grouse in grasslands managed for livestock production
Source: PLoS One. 2020 Jun 4;15(6):e0233756. doi: 10.1371/journal.pone.0233756 (PMC7272000; doi:10.1371/journal.pone.0233756)
Supplement: Attachment — Submitted filename: Response to Reviewers.pdf [file pone.0233756.s013.pdf]

Reviewer #1: Review of "Habitat selection of female" by Milligan et al.
This is well-written manuscript on a topic and species of considerable management interest. Specific comments follow, referenced by line number.

Line 14: Habitat selection affects much more than abundance, as the authors point out in the introduction. It might be helpful to add or change a word in this line to acknowledge that (something along the lines of performance, viability, or persistence).

> **Author response**: We agree and have added additional wording to convey that habitat selection affects more than just abundance (line 18).

Line 16: Consider "…thus influencing habitat selection and population size" (or performance, viability, or persistence).

> **Author response**: We have changed the wording as suggested (lines 20-21).

Line 20: Mention where the research took place.

> **Author response**: We have added the location of our study area (lines 25-26).

Line 29: I suggest deleting the word "moderate," simply stating that we want stocking rates that maintain native rangelands.

> **Author response**: We feel that it is important to include the word moderate to avoid overstating our conclusions, particularly given the number of studies that have found negative effects of high stocking rates (Briske et al. 2008, McNew et al. 2015, Kraft 2016, Winder et al. 2018). Stocking rates throughout our study area were generally moderate and it is possible that wildlife responses may be different at higher stocking rates. Therefore, we have kept the wording to qualify our conclusions.

Line 65: Here and throughout the paper, many of the cited references are about greater prairie-chickens, not sharp-tailed grouse. I realized they are congenerics and that more information is available about chickens than sharptails, but I feel like there is a bit of a bait-and-switch by mentioning sharptails in some instances and grouse (accompanied by chicken papers) in others. Many solid papers about sharptail habitat selection and reproduction are not cited in this manuscript. In addition, management, environment, and social factors differ considerably between the authors' study area and the Flint Hills, and the paper would be improved if more background was provided for the study area.

> **Author response**: We agree that it would be better to have more citations from sharp-tailed grouse papers and we have tried to include them wherever possible (e.g., Christenson 1970, Hillman and Jackson 1973, Marks and Marks 1987, Cope 1992, Saab and Marks 1992, Roersma 2001, Manzer 2004, Boisvert et al. 2005, Goddard et al. 2009, Stonehouse et al. 2015). However, there is no previous research on sharp-tailed grouse habitat selection in relation to grazing management, which is why we had to rely on the prairie-chicken literature, particularly studies in the Flint Hills, which have evaluated the relationship between grouse and grazing in depth.

Line 107: Clarification on some of the metrics would be good. The paper mentions "unfragmented grassland patches," but that's a bit of an oxymoron to me. Landscape ecologists think of habitat patches that, depending on patch size and arrangement, constitute a fragmented

or unfragmented landscape. If patchiness is determined by grazing regime, then it would be more clear to refer to heterogeneity or something similar.

> **Author response**: We agree that our use of the term "fragmented" is confusing because it is scale-dependent and grasslands are naturally heterogeneous. In this context, we were referring to grassland fragmentation caused by other land uses, such as cropland. We have clarified that we hypothesized that grouse would select for large grassland patches and removed reference to "unfragmented patches" (line 111).

Line 199: A major shortcoming to compositional analysis is that it only considers composition of the landscape and doesn't consider things like proximity to roads, wells, edge, etc. I think an RSF-based approach (similar to the next section but with a different set of availabilities) would be much more informative.

> **Author response**: We agree that one major limitation of compositional analysis is that it cannot accommodate continuous predictors. However, compositional analysis has several advantages, particularly that it assesses proportional habitat use and so avoids the unit-sum problem and that it correctly uses the individual home range as the sample unit. Although it was originally proposed in 1993, compositional analysis is still widely used in published articles today (over 50 articles citing the original paper on Google Scholar since 2019, for example). In addition, it was an appropriate approach given our objectives to evaluate home range selection relative to landcover type and grazing system, both of which are categorical variables. We have clarified both the approach and its usefulness given our objectives in the methods section (lines 214-219).

Line 214: How the available points were determined should be described, as their number and spatial distribution can substantially affect results.

> **Author response**: We have clarified that available points were sampled randomly through an individual's home range (lines 222-224).

Line 220: Perhaps "scale" instead of "spatial grain," which is frequently used to describe the resolution of landcover data.

> **Author response**: We have changed the wording throughout to use "scale" instead of "spatial grain".

Line 222: Good!

Line 234: I'm confused, as line 210 indicates that Design II of Manly et al. was used. Manly et al. states that "With design III studies the use and availability of resource units is measured separately for each animal." Random effects as espoused by reference 62 (Gillies et al.) are used when "individual animals are monitored and pooled to estimate population level effects." I'll readily admit that I'm easily confused, but I think some clarification as to what is being assessed and how it was done is necessary.

> **Author response**: We stated that we used Design III of Manly et al (2002), where the "animals in a sample are radio-collared, and the relocations of an animal identify used resource units for that animal. . . The collection of available or unused resource units within an animal's home range is sampled or censused." We have included additional language to clarify that individual telemetry locations were used locations and we

randomly sampled available locations within each individual's home range (lines 220-225).

Lines 279-280: Table 2 also confuses me, as it provides cumulative wi, but all the models (other than the null) only have one variable as evidenced by a k of 3. I suggest deleting the column with the cumulative wi or explaining the purpose of that column.

Author response: This is a good point and we have removed the column showing cumulative weights.

Lines 289-290: The "Total" row should be eliminated. Simply state that there was a total of 142 females. The other values appear to be means, except for Max area, which is not the mean but a copy of the value for rest-rotation.

Author response: We have removed the "Total" row.

Lines 292-293: The compositional analysis doesn't really provide a whole lot of information that would be helpful for management.

Author response: Given the strong selection for mixed-grass prairie, we feel that the compositional analysis provides valuable evidence for the importance of large grassland patches. We have clarified the language to stress that we mean large patches and not unfragmented patches, given that the term "fragmented" could mean multiple things in this context. This should clarify that our results suggest that conserving large grassland patches from conversion to other land uses is an appropriate and useful management strategy for the conservation of sharp-tailed grouse.

Line 302: As mentioned before, "grain size" should be replaced. Here it suggests an area of 1300m x 1300m, but in reality, 1300 m is the radius of a circle with an area 3.14 times larger than the implied square.

Author response: We have changed the wording throughout to use "scale" instead of "spatial grain".

Line 315: What about the signs of the relationships in Figure 3?

Author response: We have redone our third order analysis and now included a graph showing the mean and 95% credible intervals for each population-level selection coefficient in the analysis which shows the direction and strength of the relationships (Fig 3).

Line 319: Figure 4 indicates that females select for proximity to roads, which is quite different from the abstract, which states that females select for large intact grasslands.

Author response: Although this relationship was very weak, it was no longer significant after we reanalyzed the data following the second reviewer's comments.

Lines 348-350: Good point.

Lines 388-401: This paper focuses on habitat selection, but it would be useful if the authors provided more information from other aspects of their research regarding how reproductive success was influenced by habitat, and if the same factors popped out in both analyses.

Author response: We have included references to other aspects of our research evaluating both adult survival and reproduction to place our results in context and inform the reader how other aspects of grouse ecology were influenced by the same or different habitat variables (lines 342-344, lines 349-350, lines 371-372).

Reviewer #2: Overall, this is a nicely set-up study that seeks to answer some pertinent, long-unanswered questions about effects of livestock management on a sharp-tailed grouse and their breeding habitat. The paper is generally well written and the conclusions appear well-supported by the results. I do, however, have a couple substantial issues with the analysis and its interpretation. These concerns are related and may be cleared up fairly quickly.

First, the third-order resource selection model seems to be misspecified. Random effects seem to have been included in a way that makes no biological or mathematical sense.

Second, the considerable space is given to interpreting these random effects as 'individual variation' in behavior, which is an interpretation that the model structure—if I'm seeing it right—doesn't support.

I'm having a hard time knowing what I'm looking at in the results because the methods lack important details about the model. I would like to see the methods clarified by including full model statements (including priors—the reader is told that the priors are vague or uninformative, but has no way of verifying that for themselves because they are not provided) and, ideally, JAGS model code in Supplemental Materials.

If I am correct in my understanding that random intercepts, but not random slopes, were included in this RSF model, then I would like to see this corrected by either a) removing the random intercepts and any references to variation among individuals in their behavior, since this wasn't a feature of the model, or b) adding random slopes and interpreting those parameters as individual variation.

Option A would take hardly any time at all, whereas Option B would entail a bit of work. I'm not set on either one, but I do tend to think a random-intercept/random-slope model is more appropriate for this study design.

Author response: Thank you for the excellent review and recommendations. We have followed Option B and redone our analysis with random slopes, which allows us to interpret those parameters as individual variation. We have also included model code in the Supplemental Materials so that a reader can fully understand how the model was specified.

Line-by-line comments:
Line 25: May need to remove reference to individual-level variation in habitat selection; see detailed comments on methods below.

Author response: Given that we have now included random slopes in our model, we can make inferences regarding individual-level variation in habitat selection.

Line 39: I'm being nitpicky here, but this is such a vague, fluffy statement that it could mean

almost anything. Perhaps a specific example of how ‘conservation and management actions’ could ‘consider’ this information would imbue this statement with some needed substance.

> **Author response**: We have removed this sentence.

Line 54: Make the speculative/hypothetical nature of this claim perfectly clear here. The two provided citations provide no actual evidence that they function like patch-burn grazing. The UGBEP makes a pretty unequivocal statement that they benefit upland birds, but provides no evidence backing that up whatsoever. Frisina’s paper only deals with whether rotational grazing can reduce conflict between cattle and elk on winter range, and never mentions heterogeneity. Just make sure you don’t accidentally help propagate misinformation by leading the reader (most of whom don’t check citations) to believe that there are studies that support this.

Line 61: Same comment here as above. Yes, they will have increased residual cover, but I don’t think I’ve ever seen any evidence that RGS increase structural heterogeneity or create a ‘patchwork’ relative to other grazing systems (I haven’t searched exhaustively, though). We actually found the opposite effect in Roundup, and it looks like heterogeneity in VOR was lowest on RGS ranches in your study, too (Fig 6, random plots, in your recent REM paper--you could even cite that). You could simply add a statement like, “but this hypothesis has not been adequately tested.”

I think the purpose of RGS may have been lost in translation between range managers and wildlifers. In Bailey and Brown (2011) Rangeland Ecol Manage 64:1–9, they write, “To alleviate selective grazing across multiple scales, many rangeland managers have implemented rotational grazing systems (RGS)...The potential benefits are projected to derive from a decrease in area available to livestock at any given time (improved distribution); an increase in stock density (increased uniformity of defoliation across species and communities)...”

So, rangeland managers may be quite aware that these systems are having the exact opposite effect, at least in some settings.

> **Author response**: We have changed the wording as suggested to emphasize that this is a hypothetical relationship (line 58).

Lines 70–71: This sentence is also very vague. What kind of ‘implications?’

> **Author response**: We have changed the wording to be specific about the kind of implications (lines 73-74).

Line 185: Define an ‘abrupt change.’

> **Author response**: We have changed the wording to more clearly define how we measured edge density (lines 189-191).

Line 200: I’d like to see more detail on your methods for the second-order resource selection analysis, rather than just referring the reader to a couple citations. Which variables were considered, how was model fitting and model selection accomplished? There’s no way I could re-create your analysis with the details you give here.

> **Author response**: We have included additional details on both variables and models considered in the analyses assessing home range size and second order selection. We included additional information on which models were evaluated and our model selection procedure for the home range size analysis and we have included further details on compositional analysis to make the process more transparent (lines 181-219).

Lines 232–234: "For all models, we used generalized linear mixed models in a Bayesian framework with a logit-link and female ID as a random intercept to account for potential autocorrelation among sampling points [62, 63]."

I'm pretty sure this isn't doing what you intended. Given your particular availability sampling design, these random intercepts are unnecessary and shouldn't affect your inference at all. You later (lines 253–255) describe σ 2 as representing individual-level variation in selection for various habitat variables, which is a bit different than the way you justify inclusion of random intercepts here (autocorrelation in the response variable--not sure what that means in this case), but this is not what random intercepts do, either.

Consider what the intercept represents in a simple, univariate logit-link resource selection function where you've centered your covariate at zero. If you convert it to the probability scale, it's the predicted probability that a case/sample is a used case (1) rather than an available case (0) at the mean value of the covariate. In a use-availability RSF, its value is going to reflect the prevalence of use cases—your ratio of used to available samples. You determined that when you decided how many random samples to include for each used sample. The intercept has no biological meaning in an RSF, it is simply a reflection of the way the investigator sets up the analysis.

Unless you included both random intercepts and random adjustments to the slope (coefficient for the covariate—e.g., ,where the j subscript indexes individuals, $\beta_j \sim N(\beta, \sigma 2)$ from your citation (#69), then all you are doing by allowing the intercept to vary among individuals is allowing random adjustments to the overall (i.e., regardless of the covariate value) probability of use.

Given this is a design parameter, not a biological one, and in your study design you've fixed it at 1:15 = 0.0625 for all individuals in your dataset (this is why your y-axis tops out at about 0.07 in Fig. 4), there is no reason to expect that there will be any variation among individuals at all. By design, individuals do not vary in mean P(use) (i.e., prevalance), and you've fixed the regression coefficients, so they don't vary in their responses to covariates. So what is the variation you're modeling with these random effects? You can prove this to yourself by running this R code with your data:

```
require(here)
require(tidyr)
require(dplyr)
require(lme4)
df <- read.csv(here('data/Pts.15.FULL.csv'), stringsAsFactors = FALSE)
# check that prevalence is equal across individuals
df %>%
select(id, use) %>%
group_by(id) %>%
summarize(prevalence = mean(use)) %>%
pull(prevalence)
# scale and center continuous covariates
df.scaled <- df %>% mutate_if(is.double, scale)
# fit a simple univariate fixed-effects glm
m.fixed <- glm(use ~ 1 + roaddist, data=df.scaled, family=binomial(link = 'logit'))
# fit the same model, but with random intercepts for individuals
```

```
m.rand.intercept <- glmer(use ~ 1 + roaddist + (1|id), data=df.scaled,
family=binomial(link = ‘logit’))
# note warning about boundary effect
# check estimates and SE's of fixed effects (your inference)
summary(m.fixed)
summary(m.rand.intercept)
```

The SEs and coefficient estimates are equal for these two model specifications. Your inference is exactly the same. Also look at the estimate of individual-level variation for the glmer--it’s effectively zero (the boundary effect).

The random intercept takes on a very different role when you also include random slopes. If individuals vary in their selection for a covariate, then you need random intercepts because it doesn’t make sense to force P(use) through a single point along the x-axis. If some respond positively and others negatively to distance to roads, for example, there’s no reason to expect they’ll all converge on the exact same probability of selection at the mean distance to roads in your dataset. Random intercepts give some needed flexibility there.

Your random intercepts, however, have no clear biological interpretation that I can think of, which is probably why your results show they’re all about the same, may in fact be zero, and, if you plotted their densities instead of means and CRIs, you’d probably find them to closely reflect their priors (which I’m guessing were Uniform(0,2) based on Fig. 5). This is a really long-winded way of saying you need random slopes, not just random intercepts, if you want to know anything about individual-level variation in selection. If you don’t, you should take the random intercepts out because prevalence is a design parameter that you’ve fixed to be constant across individuals (Line 231). They are not affecting your inference.

> **Author response**: While random intercepts were necessary given our unbalanced sampling design (individuals had different numbers of relocations), we have redone this analysis to now incorporate random slopes in addition to random intercepts.

Line 243: “Regression coefficients for each variable were the product of binary indicator variables and both continuous and categorical covariates.”
Hmmm, this doesn’t make sense the way it’s worded. They should be the product of the indicator variable and an effect size parameter (the ‘raw’ coefficient). Multiplying the indicator by the covariate (the whole vector? I’m having a hard time picturing what you mean here) will give you either a vector of zeros or your original vector of covariates. It won’t give you a regression coefficient.

> **Author response**: Now that we have redone this analysis, we have removed this statement.

Line 246: “We assumed that all variables with high inclusion probability based on the posterior distributions of their indicator variables influenced habitat selection and variables with inclusion probabilities ≤ 0.25 were unimportant [67]”
Mutshinda et al. used ≤0.25 to identify unimportant variables because they used a fixed prior probability of 0.5, so that posterior probability coincides with a Bayes factor of 0.33, or 3:1 against the variable. Because you have a distribution of prior odds, rather than a fixed value, 0.25 doesn’t have any real meaning here. If, for example, your model converged on an inclusion probability of 0.1, then 0.25 would actually represent a Bayes factor of 3, or 3:1 evidence for the variable. If anything, I could see taking the median posterior inclusion probability and using that

to calculate the cutoff, but that seems a little circular, too. I'd argue that variable selection is simpler if you just pick what you think is an appropriate prior probability and consider it fixed. With the amount of data you have, I doubt this value is going to have much of an influence on which variables are identified as important, but you could always try running it with a few values to check that sensitivity. Unless mean variable inclusion probability is a parameter you're interested in making inference on (which it doesn't seem like it is, given that you don't report it in the results), then I don't quite see the value in putting a prior on it and estimating it.

> **Author response**: We have redone the analysis and are no longer using indicator variables.

Line 250: Define "standardized coefficients."

> **Author response**: We have clarified that we centered and scaled all predictor variables to calculate standardized coefficients (lines 255-257).

Line 252: So are you using credible intervals or the posterior inclusion probability to determine which variables are important? Is one given higher priority than another?

> **Author response**: We are no longer using inclusion probabilities and have clarified that we used 95% credible intervals to determine which variables were important (lines 257-258).

Line 253: I'm not exactly sure what these σ^2 are, but given that you did not include random slopes, they cannot represent individual variation in behavior.

> **Author response**: We have now included random slopes and make inferences on individual variation using the variation in individual-specific slopes for each variable (Fig 4) and the number of individuals that were selecting for and against each variable (Fig 5).

Line 259: "Vague uniform or normal priors were used for all model parameters related to covariates and their measures of error."
This statement is itself a little vague. I would like to see either a) complete model statements in your methods section or b) your JAGS model code (in supplemental info?) so I can see exactly what priors were placed on each parameter. Ideally, provide both.

> **Author response**: This is a good point and we have now included model code in our Supplemental Material so that the reader can fully understand our methods.

Line 269: Insert 'Gelman-Rubin statistic' before 'values' or just refer to them as 'potential scale reduction factors' so we know what values you're referring to.

> **Author response**: Given our new analysis approach (fitting a model with integrated nested Laplace approximation), this statistic is no longer relevant as it applies to MCMC approaches.

Line 270: I'm glad to see you assessed model fit, but it would be nice to know which specific 'attribute of the data' you used to calculate the Bayesian p -value.

> **Author response**: We have removed this statement given our new analysis approach.

Line 290: Were any models with >1 predictor considered? Again, more details on the 2nd order resource selection analysis are needed.

Author response: We have included additional details on both variables and models considered in the analyses assessing home range size and second order selection. We included additional information clarifying that only single-variable models were evaluated and our model selection procedure for the home range size analysis and we have included further details on compositional analysis to make the process more transparent (lines 181-219).

Lines 292–293: This sentence and Table 3 seem to provide exactly the same information, so I would just include one or the other. I find Table 3 hard to interpret (isn't the info on either side of the diagonal redundant? And why isn't there a #2-ranked landcover?), so I'd suggest axing it.

Author response: This is a good point and we have now removed Table 3.

Line 308: Again, I don't think 0.25 is a meaningful cutoff. Try to come up with a criterion more applicable to your particular model.

Author response: With our revised analysis, we are no longer using indicator variables and so no longer require a cutoff.

Line 311: This doesn't indicate individual variation in habitat selection unless you fit random intercept and random slope models. So am I right to assume these variances indicate variation among individual-level adjustments to the intercept? If so, why is there more than one? Are these from separate, univariate models? I'm generally very confused about your model structure, so I'll reiterate here that it would really help if you provided model statements and code so readers know exactly what parameters these are. Also (and this is a personal preference thing unless the journal has specific guidelines about it), I'm much more accustomed to thinking of variation on the scale of standard deviations rather than variances. Seems easier to interpret since it's on the same scale as the regression coefficients and data. Is there a specific reason you report σ 2 rather than σ?

Author response: Now that we have included random slopes in our model, we make inferences about individual variation using the variation in individual-specific slopes for each variable (Fig 4). We have also included model code in our Supplemental Material so readers know exactly how the model was specified.

Line 377: Given that you have the ability to fit random-slope, random-intercept models (because you sampled availability at the individual level), there is no reason you shouldn't. In fact, I would argue that assuming the slopes are fixed across individuals is inappropriate given that availability differed across your study area, as you state on Line 348. At the individual level and the third-order, you are only likely to detect avoidance of, say, oil and gas features, if there are oil and gas features within the home range to avoid. By forcing all the responses to be the same among individuals, you're not allowing your models to pick up on those potential functional responses to availability. Moreover, fitting random-slope, random-intercept models does not preclude making inference at the population level. The beauty of these multi-level models is that you can make inference at multiple levels of the hierarchy—population or individual. Going back to the example of citation #69, this model provides not only estimates of the individual-level β j 's, but also the population-level β .

Author response: This is a good point and we have now included random slopes.

Fig. 1. Might this look a little better on the log(ha) scale?

Author response: We feel it is more intuitive if the variable is not transformed, but can defer to the associate editor.

Fig. 2. Since both variables are continuous, why not plot the data? It's a more honest way of showing the strength and predictive capability of a relationship than just plotting the regression line for the mean and its confidence interval.

Author response: We have now included the raw data in our plot of the regression results (Fig 2).

Fig. 3 and Fig. 5: Both of these figures depict information that seems better suited to a table. Just a suggestion.

Author response: We have redone our analysis, so Fig 3 and Fig 5 now present different information.
