# Supplementary material for: Habitat selection of female sharp-tailed grouse in grasslands managed for livestock production
Source: PLoS One. 2020 Jun 4;15(6):e0233756. doi: 10.1371/journal.pone.0233756 (PMC7272000; doi:10.1371/journal.pone.0233756)
Supplement: Attachment — Submitted filename: milligan sharptail R1 review.pdf [file pone.0233756.s014.pdf]

Overall, I'm very happy with the improvements that were made in response to the first round of reviews. I'm especially glad the authors chose to re-analyze the third-order resource selection data with a varying-intercept and varying-slope model. There are just a few minor interpretation issues that I think should be addressed. These are discussed below.

Line-by-line comments on PONE-D-19-33753R1:

Line 190: This is a little too vague to reproduce. Is it edge length divided by home range size?

Line 209: You might consider using a term other than 'home range' in this context, since it doesn't really apply to a group of individuals. Maybe '95% utilization distribution' or something like that.

Line 275: I didn't catch this before, but you must have had several females that contributed multiple home ranges to this analysis. In keeping with the rest of your analysis, you should account for this non-independence with random effects or subsample so each individual contributes just one home range.

Figure 1: Point taken re: interpretability, and I'll defer to the AE's judgement on this, too, but it's awfully hard to see any differences among the means on this scale.

Figure 2: Need units on the x-axis label (m/km^2, or whatever these are).

Line 308: Strike 'the variable of'.

Figure 3: The y-axis label could be a lot more informative than 'effect size.' Maybe, 'population level selection coefficient,' or 'global mean selection coefficient.'

Figure 4: Comparing this figure ot Figure 3, it's apparent that these are not the individual-level slopes per se, but the individual-level adjustments to the population-level means shown in Figure 3. This should either be explained more clearly in the figure caption, or (and I'd prefer this) you could show boxplots of the actual individual-level slope estimates. This would show the location and scale of these slope parameters in one place.

Line 313 and Figure 5: This figure, and Figure 4, are interesting in that they show there is substantial individual-level variation in selection *coefficients*. But they immediately raise the question: Are we actually seeing individuals that vary widely in their habitat preferences, or are we just seeing more-or-less constant 3rd-order habitat use among home ranges with highly variable resource availability (i.e., functional responses, *sensu* Mysterud and Ims)? You could answer that with your data, but you kind of leave us hanging. And the statement on Line 313 that you're seeing 'no population-level selection' kind of sweeps this issue under the rug.

Line 329: What you've shown here hasn't convince me we are seeing 'highly plastic habitat use.' Keep in mind the distinction between resource use and resource selection. You've demonstrated highly variable selection coefficients, but you have not shown that individuals are highly variable in what they're actually using. This comes back to my comment above re: functional responses to availability. Because you don't test for functional responses, you haven't convinced me these birds are actually displaying divergent behaviors/preferences when it comes to third order resource selection.

I'm not saying you're interpreting your data wrong, just that you haven't quite provided us, the readers, with enough evidence. I went ahead and plotted mean use (y-axis) against mean availability (x-axis) for several of your variables among home ranges, and this is what it looks like:

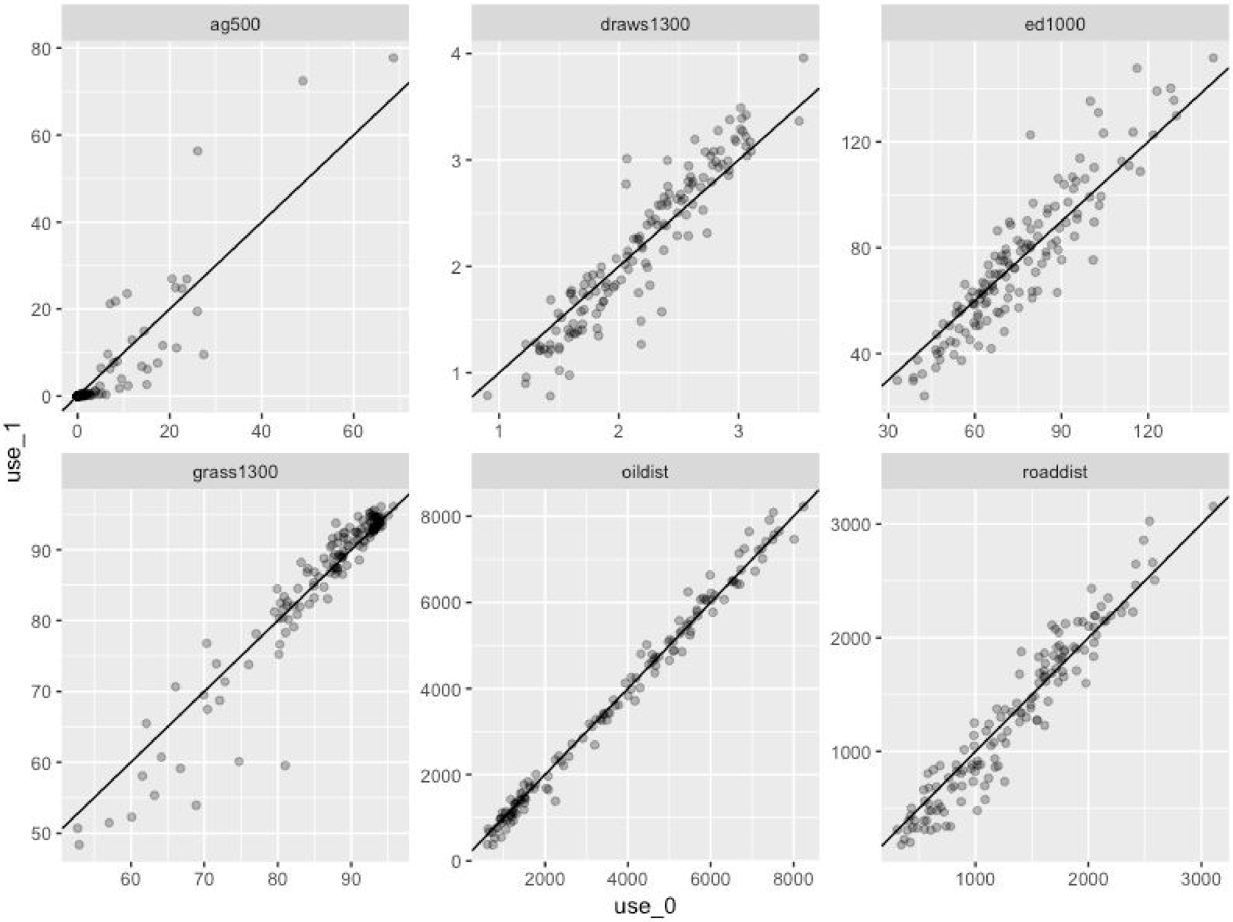

This tells me you're probably right, they are very plastic in what they use. In fact, use seems to be more variable than availability in some cases. I'd suggest including something like this, or (even better) selection coefficients plotted against mean availability, if you want to make your argument of highly plastic habitat preference/use stronger. You could even combine the

information in Figure 5 with this type of plot by color coding the home ranges according to positive, null, or negative individual-level selection coefficients.

Line 351: This sentence seems like a holdover from the last version of the manuscript, before you incorporated individual-level random slopes. Given what the plot above shows, I don't think variation in availability among home ranges is obscuring some real response to oil pad distance—they just don't seem to be avoiding them at all.

Line 361: Reconsider the word 'inconsistent' here; there's nothing inconsistent about finding different habitat relationships for different species in a different ecological contexts.

Line 376: More important than what? I'm not picking up on your meaning here.

Line 380: This sentence also reads like a holdover from your last version.

Line 386: Clarify that you mean individual differences in resource selection. As I read your suggestion for future research here, it seems even more critical that you show readers that these differences in selection coefficients are not simply functional responses to availability. If they are, then your suggestion that they represent alternative strategies is much weaker.

Line 401: It's up to you how much you want to synthesize the results of your various analyses for the readers of this paper, but I can't help but think you're leaving out some critical knowledge from your last paper in this conclusion. Just reading this, I'm left with the impression that grazing systems are irrelevant to sharp-tailed grouse in this ecosystem. But what about the fact that you showed nest success was significantly lower in these rotational grazing systems? If they're not avoiding them, but they're achieving lower reproductive success in them, then RGS aren't just neutral or not quite as good as we'd hoped, they're a (potentially) bad management practice for sharp-tailed grouse!

Again, this is largely a matter of personal preference, but I would personally love to see some synthesis of what you're finding out there. Don't assume people will search out all your research and put the puzzle together themselves.

Table S2: This table title should probably include more detail so it can stand alone. Something like: "Support for models predicting the spatial grain of each landcover variable that best predicts third-order female sharp-tailed grouse habitat selection during the breeding season, 2016–2018, based on deviance information criteria (DIC) values.

Table S3: Same comment as above.
