# Supplementary material for: Habitat selection of female sharp-tailed grouse in grasslands managed for livestock production
Source: PLoS One. 2020 Jun 4;15(6):e0233756. doi: 10.1371/journal.pone.0233756 (PMC7272000; doi:10.1371/journal.pone.0233756)
Supplement: Attachment — Submitted filename: Response to Reviewers.docx [file pone.0233756.s015.docx]

Additional Editor Comments (if provided):

We thank the authors for addressing the reviewer comments in great detail. I would request the authors address the remaining concerns of the same 2 authors as well as my own concerns regarding second-order selection.

In the previous draft, a reviewer requested more details on second-order selection, particularly on the variables and reason for not conducting RSF for second-order as done with third-order. I don’t believe the authors adequately addressed the previous concerns but more importantly the results seem confusing to me. Let’s start with the justification and the response from the authors in the previous version:

Line 200: I’d like to see more detail on your methods for the second-order resource selection
analysis, rather than just referring the reader to a couple citations. Which variables were
considered, how was model fitting and model selection accomplished? There’s no way I could
re-create your analysis with the details you give here.

Author response: We have included additional details on both variables and models considered in the analyses assessing home range size and second order selection. We included additional information on which models were evaluated and our model selection procedure for the home range size analysis and we have included further details on compositional analysis to make the process more transparent (lines 181-219).

AE’s comments: The authors state on Lines 210: “We used compositional analysis to compare used versus available landcover types and grazing systems separately.” However, in the results the authors identify results only for landcover types (Lines 295-301) in the order of preference. They then claim in Lines 301-303 that “There was no evidence that selection of home ranges in relation to grazing system was different from random (p = 0.20), suggesting that females were not differentiating between pastures in the different grazing systems.” This appears to be relating the linear models in the preceding section linking size of home range to grazing system, not compositional analysis? If it were a separate analysis on grazying system, I would expect a similar sentence of the 3 systems as presented for the landcover types in Lines 295-301 (i.e., no preference for grazing system). I am not sure if I am missing something here?

More importantly, your justification for second-order selection that “others are still using it” is poor and I recommend the authors delete the entire concept of second order selection in your manuscript. Although the authors present compositional analysis in the Methods and Results, the authors then combine both second- and third-order selection in a statement in Lines 357-358 and second-order selection is never mentioned again. The details of second-order selection does not add much obvious value to the manuscript and the authors do not even spend any time on its contribution in the Discussion?

**Author response:** Thank you for these comments. We believe that it is important to include both second and third order selection because these analyses evaluate habitat selection at different spatial scales, but we have followed the first reviewer’s recommendation and changed our second order analysis so that it uses resource selection functions similar to the third order analysis rather than compositional analysis. This has allowed us to include additional continuous variables, such as distance to oil pad, which could not be accommodated with our previous analysis approach. We have outlined our approach in detail and believe that it adds important information to the manuscript.

Reviewer #1

Lines 32-33: Very minor point of subject-verb agreement: “…strategies…are important for the conservation of sharp-tailed grouse. “

**Author response:** We have corrected this sentence (Line 33).

Line 69: Similar to a comment in my initial review, I find this type of citation to be misleading. Sharp-tailed grouse are “Recognized as an indicator species for grassland ecosystems” by an unpublished master’s thesis from Kansas on lesser prairie chickens? Yes, sharptails are recognized as indicator/keystone/umbrella species, but there are certainly more appropriate entities to cite, including state wildlife management plans, joint venture implementation plans, and other plans developed by groups involved with on-the-ground conservation in the study area.

**Author response:** This citation refers to a thesis on sharp-tailed grouse in Alberta that provided the best empirical evidence of which we are aware regarding sharp-tailed grouse as indicator species (Line 69). We agree that there are other plans that cite sharp-tailed grouse as indicator species, but they did not evaluate this concept, which is why we chose this citation. We can provide additional citations if the editor feels that is necessary.

Lines 218-219: The objective might have been to evaluate landcover type and grazing system, but in many areas those are influenced by or correlated with continuous variables such as proximity to road, proximity to water, or topographic variation, to name a few. I still maintain that the compositional analysis leaves out important information. Variables quantifying anthropogenic disturbance are included in the within-home-range analysis (line 227); it only makes sense that if birds avoid such disturbance within their home range that they might avoid such disturbance when selecting a home range.

**Author response:** Following this recommendation, we have redone our second order analysis and now use resource selection functions which evaluate all the variables included in the within-home range analysis.

Line 289: Final decision is up to the editor, but most people find informative figure headings easier to understand than descriptive, i.e., the heading for Figure 2 would convey more information and be easier to assimilate if it read “Home range size was negatively related to density of edge habitat within the home range.” Other headings could be similarly revised.

**Author response:** We can defer to the editor on this.

Lines 349-352: But habitat selection across the landscape did not consider these factors, and the reason that some individuals had few to no roads or oil wells within their home range might be because they selected areas with few to no roads.

**Author response:** Our second order analysis now includes these continuous variables, including roads and oil pads, so we have updated our discussion to reflect this.

Lines 371-373: It was stated earlier that topography and soil type didn’t vary among grazing systems, but if there was substantial variation across the landscape (regardless of grazing system), might they have affected habitat selection?

**Author response:** We have revised our analysis, so that it now includes additional variables which altered our discussion, so we have removed this sentence.

Reviewer #2: Overall, I'm very happy with the improvements that were made in response to the first round of reviews. I'm especially glad the authors chose to re-analyze the third-order resource selection data with a varying-intercept and varying-slope model. There are just a few minor interpretation issues that I think should be addressed. These are discussed below.

Line-by-line comments on PONE-D-19-33753R1:

Line 190: This is a little too vague to reproduce. Is it edge length divided by home range size?

**Author response:** We have clarified that edge density was edge length divided by home range size (Lines 196-198).

Line 209: You might consider using a term other than ‘home range’ in this context, since it doesn’t really apply to a group of individuals. Maybe ‘95% utilization distribution’ or something like that.

**Author response:** This is a good point and we clarified that the study area was defined by a utilization distribution, which is a more appropriate term than home range (Lines 205-206).

Line 275: I didn’t catch this before, but you must have had several females that contributed multiple home ranges to this analysis. In keeping with the rest of your analysis, you should account for this non-independence with random effects or subsample so each individual contributes just one home range.

**Author response:** We have updated our second order analysis and we only included one home range from each individual, which we clarified in the methods section (Lines 202-203).

Figure 1: Point taken re: interpretability, and I’ll defer to the AE’s judgement on this, too, but it’s awfully hard to see any differences among the means on this scale.

**Author response:** We can defer to the editor on this, but we found no evidence for a difference in home range size among grazing systems, which is what is depicted in this graph.

Figure 2: Need units on the x-axis label (m/km2, or whatever these are).

**Author response:** We have included units on the x-axis.

Line 308: Strike ‘the variable of’.

**Author response:** We have removed this phrase.

Figure 3: The y-axis label could be a lot more informative than ‘effect size.’ Maybe, ‘population level selection coefficient,’ or ‘global mean selection coefficient.’

**Author response:** We have revised the y-axis label following this recommendation.

Figure 4: Comparing this figure to Figure 3, it’s apparent that these are not the individual-level slopes per se, but the individual-level adjustments to the population-level means shown in Figure 3. This should either be explained more clearly in the figure caption, or (and I’d prefer this) you could show boxplots of the actual individual-level slope estimates. This would show the location and scale of these slope parameters in one place.

**Author response:** We have fixed this figure so that it now shows boxplots of the actual individual-level slope estimates.

Line 313 and Figure 5: This figure, and Figure 4, are interesting in that they show there is substantial individual-level variation in selection coefficients. But they immediately raise the question: Are we actually seeing individuals that vary widely in their habitat preferences, or are we just seeing more-or-less constant 3rd-order habitat use among home ranges with highly variable resource availability (i.e., functional responses, sensu Mysterud and Ims)? You could answer that with your data, but you kind of leave us hanging. And the statement on Line 313 that you’re seeing ‘no population-level selection’ kind of sweeps this issue under the rug.

**Author response:** This is a good point and we have included an additional figure (Figure 6) to allow readers to better evaluate our conclusions and to demonstrate that we are seeing highly flexible habitat selection, not a functional response.

Line 329: What you’ve shown here hasn’t convince me we are seeing ‘highly plastic habitat use.’ Keep in mind the distinction between resource use and resource selection. You’ve demonstrated highly variable selection coefficients, but you have not shown that individuals are highly variable in what they’re actually using. This comes back to my comment above re: functional responses to availability. Because you don’t test for functional responses, you haven’t convinced me these birds are actually displaying divergent behaviors/preferences when it comes to third order resource selection.

I’m not saying you’re interpreting your data wrong, just that you haven’t quite provided us, the readers, with enough evidence. I went ahead and plotted mean use (y-axis) against mean availability (x-axis) for several of your variables among home ranges, and this is what it looks like:

[see pdf version of comments!]


This tells me you’re probably right, they are very plastic in what they use. In fact, use seems to be more variable than availability in some cases. I’d suggest including something like this, or (even better) selection coefficients plotted against mean availability, if you want to make your argument of highly plastic habitat preference/use stronger. You could even combine the information in Figure 5 with this type of plot by color coding the home ranges according to positive, null, or negative individual-level selection coefficients.

**Author response:** Thank you for the useful suggestion. We have included a figure (Figure 6) like the one you suggested that plots resource use against availability and combined it with the previous Figure 5 to show the number of individuals selecting for and against specific habitat variables. This figure includes important information about the variation in individual selection and is similar to previously published figures evaluating functional responses.

Line 351: This sentence seems like a holdover from the last version of the manuscript, before you incorporated individual-level random slopes. Given what the plot above shows, I don’t think variation in availability among home ranges is obscuring some real response to oil pad distance—they just don’t seem to be avoiding them at all.

**Author response:** We have revised our discussion and removed this sentence.

Line 361: Reconsider the word ‘inconsistent’ here; there’s nothing inconsistent about finding different habitat relationships for different species in a different ecological contexts.

**Author response:** Given our updated results, we have revised this sentence (Line 368).

Line 376: More important than what? I’m not picking up on your meaning here.

**Author response:** We have revised our discussion and removed this sentence.

Line 380: This sentence also reads like a holdover from your last version.

**Author response:** We have removed this sentence.

Line 386: Clarify that you mean individual differences in resource selection. As I read your suggestion for future research here, it seems even more critical that you show readers that these differences in selection coefficients are not simply functional responses to availability. If they are, then your suggestion that they represent alternative strategies is much weaker.

**Author response:** We have clarified that we mean individual differences in resource selection (Line 399).

Line 401: It’s up to you how much you want to synthesize the results of your various analyses for the readers of this paper, but I can’t help but think you’re leaving out some critical knowledge from your last paper in this conclusion. Just reading this, I’m left with the impression that grazing systems are irrelevant to sharp-tailed grouse in this ecosystem. But what about the fact that you showed nest success was significantly lower in these rotational grazing systems? If they’re not avoiding them, but they’re achieving lower reproductive success in them, then RGS aren’t just neutral or not quite as good as we’d hoped, they’re a (potentially) bad management practice for sharp-tailed grouse!

Again, this is largely a matter of personal preference, but I would personally love to see some synthesis of what you’re finding out there. Don’t assume people will search out all your research and put the puzzle together themselves.

**Author response:** This is a good suggestion and we have incorporated findings from previous research to better synthesize our results (Lines 370-373 and 404-406).

Table S2: This table title should probably include more detail so it can stand alone. Something like: “Support for models predicting the spatial grain of each landcover variable that best predicts third-order female sharp-tailed grouse habitat selection during the breeding season, 2016–2018, based on deviance information criteria (DIC) values.

**Author response:** We have altered the title to be more informative following this suggestion.

Table S3: Same comment as above.

**Author response:** We have altered the title to be more informative following this suggestion.
